# Growth Hormone Alleviates Atherosclerosis Through Regulating the Activity of PI3K/AKT Pathway: Insights From Single‐Cell Sequence and Mechanism Exploration

**DOI:** 10.1155/ijog/9710652

**Published:** 2025-10-13

**Authors:** Jin Cai, Shuang Shi, Yuhang Wang, Xiangdong Zhang, Xinghua Wei, Yanjing Wu, Yunlong Shi, Bin Li, Daorong Hou, Songyun Zhao

**Affiliations:** ^1^ Department of Pediatrics, Affiliated Hospital of Nantong University, Nantong, China, ahnmc.com; ^2^ Department of Pediatrics, Medical College of Nantong University, Nantong, China; ^3^ Key Laboratory of Model Animal Research, Animal Core Facility, Nanjing Medical University, Nanjing, China, njmu.edu.cn

**Keywords:** atherosclerosis, growth hormone, inflammatory responses, oxidative stress, PI3K/AKT signaling pathway

## Abstract

**Purpose:**

This research sought to investigate the impact and underlying mechanisms of growth hormone (GH) on atherosclerosis (AS) based on the analysis of single‐cell RNA sequencing (scRNA‐seq) data.

**Methods:**

We analyzed the impact of GH on arterial vascular smooth muscle cells (VSMCs) by utilizing scRNA‐seq data obtained from both atherosclerotic and healthy vascular tissues in mice. AS was induced in C57BL/6 and ApoE^−/−^ mice through hypophysectomy performed via the parapharyngeal approach, followed by a high‐fat diet (HFD), resulting in the C57‐Hx and ApoE^−/−^‐Hx models. AS was evaluated by measuring arterial lipid deposition, plaque progression, collagen loss, vascular inflammation, and oxidative stress. Serum metabolite alterations were assessed using liquid chromatography‐mass spectrometry (LC‐MS). RNA sequencing was employed to examine the underlying mechanisms of GH in the context of AS treatment, with findings further confirmed through western blot analysis. In vitro experiments involved treating VSMCs with oxidized low‐density lipoprotein (ox‐LDL) to simulate atherosclerotic injury. The formation of foam cells was evaluated by measuring lipid accumulation, inflammatory responses, apoptosis, and the expression levels of foam cell‐related markers. Finally, the PI3K/AKT inhibitor LY294002 confirmed that GH alleviates AS via the PI3K/AKT signaling pathway.

**Results:**

scRNA‐seq data analysis showed that growth hormone signaling was reduced in VSMCs of atherosclerotic arteries. HFD led to elevated levels of serum total cholesterol (TC), triglycerides (TGs), and low‐density lipoprotein cholesterol (LDL‐C), accompanied by increased lipid deposition, inflammatory responses, and oxidative stress. In contrast, high‐density lipoprotein cholesterol (HDL‐C) and insulin‐like growth factor 1 (IGF‐1) levels were lower in C57‐Hx mice. GH treatment improved HFD‐induced AS in ApoE^−/−^‐Hx mice. LC‐MS analysis revealed that GH altered lipid metabolism in serum samples from C57‐sham, C57‐Hx, ApoE^−/−^‐Hx, and ApoE^−/−^‐Hx‐GH(3) mice. GH maintained lipid balance by increasing 1‐palmitoyl‐2‐oleoyl‐sn‐glycerol‐3‐phosphocholine (POPC), PE‐NMe2(18:1(9z)/18:1(9z)) (DMPE), and 4‐chloro‐2‐nitrobenzylalcohol levels and decreasing 1‐heptadecanoyl‐sn‐glycerol‐3‐phosphocholine. RNA sequencing showed significant gene expression differences in the aortas of C57‐sham and C57‐Hx mice. Kyoto Encyclopedia of Genes and Genomes (KEGG) pathway analysis revealed that GH inhibited the progression of AS by modulating the phosphatidylinositol 3‐kinase/protein kinase B (PI3K/AKT) signaling pathway, a finding that was validated through western blotting. Further in vitro studies demonstrated that GH exerted protective effects on VSMCs against ox‐LDL‐induced damage through activation of the PI3K/AKT pathway, as evidenced by experiments using the specific PI3K inhibitor LY294002.

**Conclusion:**

GH alleviates the development of AS through the activation of the PI3K/AKT pathway. The findings of this research emphasize the therapeutic potential of GH in inhibiting AS and highlight the importance of the PI3K/AKT pathway as a promising target for clinical intervention.

## 1. Introduction

Atherosclerosis (AS) stands as the primary contributor to global cardiovascular disease mortality [1]. It is a complex, multifactorial condition that arises within the arterial walls [2]. AS is characterized by the progression of vascular lesions, starting from early fatty streaks and advancing to more complex plaques. The development of advanced plaques is marked by arterial intimal thickening, infiltration of inflammatory cells and VSMCs, accumulation of extracellular lipids (known as atheroma), and the formation of fibrous tissue [3]. Atherosclerotic plaques, the most clinically significant feature of AS, which display prominent lipid deposition and infiltration of immune cells [4]. Chronic inflammation and oxidative stress in the arterial environment contribute to the development of atherosclerotic plaques and blood clots, adding complexity to the management of AS [5].

The pituitary gland is essential in controlling cholesterol metabolism across species, including both animals and humans [6–8]. In rodent models, hypophysectomy (Hx) leads to a shift in the serum lipoprotein pattern, transitioning from primarily high‐density lipoprotein (HDL) to low‐density lipoprotein (LDL) dominance [9–11]. Animals subjected to Hx display a significant decrease in their ability to resist dietary cholesterol [10], and when exposed to a high‐fat and high‐cholesterol diet, they are more prone to developing AS [12]. Beyond GH, the pituitary also releases several other hormones, including thyroid‐stimulating hormone (TSH), follicle‐stimulating hormone (FSH), adrenocorticotropic hormone (ACTH), luteinizing hormone (LH), melanocyte‐stimulating hormone (MSH), and prolactin (PRL). Some of these hormones have been linked to a heightened risk of AS through various mechanisms, such as disrupting lipid homeostasis, promoting vascular inflammation, and contributing to plaque instability [13–16]. PRL may exert protective effects against AS by modulating cholesterol metabolism and suppressing inflammatory responses [17]. Overall, pituitary hormones play diverse roles in the progression of atherosclerotic plaques. Although Hx leads to deficiencies in multiple pituitary hormones, infusion of human or bovine GH alone into Hx mice and rats can restore lipid metabolism to normal levels [18] and reverse the elevation of atherosclerotic biomarkers [19].

Derived from the anterior pituitary, GH is a 22‐kDa peptide hormone that exerts broad regulatory effects on metabolism, growth, and systemic energy utilization [20]. Beyond its well‐documented functions in tissue development and metabolic regulation, GH also exerts notable effects on immune system activity [21]. The GH‐IGF‐1 axis is central to the development and maturation of multiple organs and works in concert to control the growth and physiological functions of various tissues [22]. Evidence from clinical research suggests that adult‐onset growth hormone deficiency (GHD) contributes to elevated cardiovascular risk and higher mortality, highlighting its importance in cardiovascular pathophysiology [23]. Moreover, GHD has been linked to a greater susceptibility to AS [24]. These observations suggest that GH may have a significant impact on lipid homeostasis and the progression of AS, although the underlying mechanisms remain incompletely defined. Here, we demonstrate that GH attenuates atherosclerotic development through activation of the PI3K/AKT pathway in vascular smooth muscle cells (VSMCs). Collectively, these findings identify GH as a potential therapeutic modulator in AS and highlight the PI3K/AKT signaling axis as a clinically relevant target for intervention.

## 2. Materials and Methods

### 2.1. scRNA‐seq Data Analysis

scRNA‐seq data from mouse aortic tissues under both normal and atherosclerotic conditions was retrieved from the Gene Expression Omnibus (GEO) repository (accession ID: GSE239591). The dataset consisted of unprocessed UMI count data from four whole‐aorta specimens: two control samples (CR1 and CR2) collected from mice fed a standard diet and two AS‐induced samples (AS1 and AS2) obtained from animals placed on a high‐fat, high‐cholesterol diet after administration of PCSK9 AAV8. All experimental animals were 8‐week‐old p16^TdTomato^+/‐ transgenic mice (*Mus musculus*, mixed sex), which received a retro‐orbital injection of 3 × 10^10^ viral genome copies of PCSK9 AAV8. The control group remained on a regular chow diet throughout the experimental period, whereas the atherosclerotic group was transitioned to a high‐fat, high‐cholesterol diet (comprising 42% fat and 0.2% cholesterol, designated as TD.88137), 2 weeks after viral delivery. After 16 weeks of dietary treatment, the entire aortas were excised and used for scRNA‐seq library construction.

The raw count matrices were processed and analyzed employing the Seurat package (Version 4.3.0) in the R programming environment. Only cells meeting the following quality filters were included for downstream analysis: number of detected genes between 200 and 6000, mitochondrial gene content below 20%, and hemoglobin gene proportion under 3%. The data underwent normalization, followed by selection of variable genes, data scaling, and dimensionality reduction via principal component analysis (PCA). To address technical variability across samples, batch effects were adjusted using the Harmony algorithm based on the Top 30 principal components. Clustering analysis was performed on the corrected embeddings, and the results were visualized using UMAP.

Potential doublets were detected and excluded using the DoubletFinder algorithm. Cell types were determined according to the expression profiles of known marker genes. The activation level of the GH signaling pathway was assessed using the AddModuleScore function in Seurat, incorporating the following gene markers: *Gh*, *Ghr*, *Igf1*, *Jak2*, *Stat5a*, and *Stat5b*. Differences in pathway activity between the control and atherosclerotic groups were evaluated specifically in smooth muscle cells using the Wilcoxon rank‐sum test.

### 2.2. Animals

Then, 8‐week‐old male C57BL/6 mice were acquired from Nanjing Medical University, while male ApoE^−/−^ mice of the same age were obtained from Gempharmatech Biotechnology Co., Ltd. (Nanjing, China). The animals were maintained in a specific pathogen‐free (SPF) environment at the Animal Core Facility of Nanjing Medical University, under controlled temperature conditions and a 12‐h light–dark cycle, with unrestricted access to food and water. Prior to the initiation of experiments, all mice underwent a 1‐week acclimatization period.

#### 2.2.1. Trial 1

The C57BL/6 mice were allocated into two distinct groups:

• C57‐Hx group: C57BL/6 mice underwent Hx via the parapharyngeal route [25] and were subsequently fed a HFD (SY19004, Shuyu Biotechnology Co., Ltd., Shanghai, China) for 10 months.
• C57‐sham group: C57BL/6 mice underwent sham operation and were subsequently fed the same HFD for 10 months.

The HFD contained 40% of calories from fat, 1.25% cholesterol, and no cholic acid. This diet is known to induce AS in ApoE^−/−^ mice for 8 weeks.

#### 2.2.2. Trial 2

The ApoE^−/−^ mice were categorized into four separate groups:

• ApoE^−/−^‐Hx + GH (1) group: ApoE^−/−^ mice that underwent Hx were fed a HFD and received daily intraperitoneal injections of GH (1 mg/kg/day, S20050024, Changchun GeneScience Pharmaceutical Co., Ltd., China) [26] for 12 weeks.
• ApoE^−/−^‐Hx + GH (2) group: ApoE^−/−^ mice that underwent Hx, were fed a HFD, and administered daily intraperitoneal injections of GH at a dose of 2 mg/kg/day over a 12‐week period.
• ApoE^−/−^‐Hx + GH(3) group: ApoE^−/−^ mice that underwent Hx, were fed a HFD, and administered daily intraperitoneal injections of GH at a dose of 3 mg/kg/day over a 12‐week period.
• ApoE^−/−^‐Hx group: ApoE^−/−^ mice underwent Hx, were fed HFD, and administered daily intraperitoneal injections of an equal volume of 0.9% normal saline over a 12‐week period.

All experimental procedures and animal handling methods followed the standards set forth in the “Guide for the Care and Use of Laboratory Animals” (National Academic Press, United States, 1996). The research involving animals was reviewed and approved by the Institutional Animal Care and Use Committee at Nanjing Medical University (Approval Number: IACUC‐2408064).

### 2.3. Whole Aorta Oil Red O (ORO) Staining

Aortas collected from each group were processed for ORO staining (G1050, Servicebio, China). Briefly, the complete aorta, extending from the aortic root to the iliac bifurcation, was carefully removed, cleared of perivascular fat, and split open along its longitudinal axis. The aortas were then laid flat, stained with ORO solution, and rinsed successively in 60% isopropanol followed by distilled water. The total surface area of the aorta and the ORO‐stained lesion area were measured using Image‐Pro Plus 6.0 software. Lesion severity was determined by calculating the proportion of ORO‐positive area relative to the entire aortic surface area, expressed as a percentage.

### 2.4. Biochemical Analysis

Blood samples were obtained from overnight‐fasted mice through cardiac puncture and left to coagulate at ambient temperature for 30 min. Following centrifugation at 1300 × *g* for 10 min at 4°C, serum was collected. The concentrations of TC, HDL, LDL, and TG in the serum were analyzed using an automated biochemical analyzer (Model 7100, Hitachi, Japan) in conjunction with commercial assay kits (TC: H202, HDL: H203T, LDL: H207, and TG: H201; MedicalSystem, China). Serum IGF‐1 levels were quantified spectrophotometrically using a commercially available kit (E‐EL‐M3006, Elabscience, China). The levels of interleukin‐6 (IL‐6; E‐EL‐M0044, Elabscience, China); interleukin‐10 (IL‐10; E‐EL‐M0046, Elabscience, China); and tumor necrosis factor‐alpha (TNF‐*α*; E‐EL‐M3063, Elabscience, China) in VSMCs were assessed using enzyme‐linked immunosorbent assay (ELISA) kits, following the manufacturer’s recommended protocols.

### 2.5. Histology and Immunofluorescence Staining

Serial sections (5‐*μ*m thick) of the aortic root were subjected to ORO (G1015, Servicebio, China), hematoxylin and eosin (HE, G1005, Servicebio, China), and Picrosirius Red (PSR, G1078, Servicebio, China) staining. For immunofluorescence analysis, adjacent sections were incubated with primary antibodies targeting CD68 (ab53444; Abcam, United States). Nuclear staining was performed using 4 ^′^,6‐diamidino‐2‐phenylindole (DAPI, MBD0015, Sigma, United States). The proportion of positive staining relative to the total plaque area was analyzed using Image‐Pro Plus software (Version 6.0), with lesion severity represented as the percentage of the positive area in relation to the entire plaque region.

### 2.6. Immunohistochemical Staining

For immunohistochemical analysis, the sections were incubated for 30 min in a blocking solution consisting of 5% goat serum diluted in DPBS supplemented with 0.1% Tween‐20 (P1379, Sigma‐Aldrich, United States) and 0.5% BSA (A1597, Sigma‐Aldrich, United States). Following this, the sections were incubated overnight at 4°C with primary antibodies specific to nitrotyrosine (sc‐71007, Millipore, United States) and TNF‐*α* (ab300093, Abcam, United States), prepared according to the recommended dilutions. All sections were incubated under identical conditions, including the same antibody concentrations and incubation times, to ensure comparable immunostaining across different experimental groups. Tissue sections were examined and captured using a light microscope and semiquantitatively analyzed with Image‐Pro Plus 6.0 software.

### 2.7. Reactive Oxygen Species (ROS) Staining

To detect total ROS in freshly frozen aortic vascular tissues, dihydroethidium (DHE, D‐23107; Invitrogen, United States) staining was performed. In brief, abdominal aortic segments were embedded in OCT compound (3801480, Leica, Germany) and stored at −80°C. The frozen tissues were then cut into 5‐*μ*m sections using a cryostat and allowed to air dry. Following this, the sections were incubated with 5 *μ*M DHE solution, prepared by diluting DHE (United States) in PBS, and incubated in the dark at 37°C for 30 min. Afterward, the sections were washed twice with cold PBS and immediately visualized under a fluorescence microscope.

### 2.8. In Situ TUNEL Fluorescence Staining Assay

The TUNEL staining procedure was carried out following the manufacturer’s guidelines (11684817910, Roche, Switzerland). Abdominal aortic tissues were fixed overnight in 4% paraformaldehyde (PFA), processed for dehydration, embedded in paraffin, and sectioned into 5‐*μ*m‐thick slices. These sections were then placed on polylysine‐coated glass slides. TUNEL‐positive cells were labeled in red, while the nuclei were counterstained with DAPI to help identify the cell type. The proportion of TUNEL‐positive cells within a single field was calculated as an indicator of the apoptotic cell death rate.

### 2.9. Serum Metabolite Profiles

Serum metabolites were analyzed using an ultra‐high‐performance liquid chromatography (UPLC) system (LC‐30A, Shimadzu, Japan) interfaced with a TripleTOF 6600+ mass spectrometer (SCIEX, United States). Metabolite identification was achieved by automated comparison of retention times, ion characteristics, and tandem mass spectrometry fragmentation profiles. Only compounds with a combined score of ≥ 0.5 and a coefficient of variation (CV) from quality control (QC) samples below 0.3 were selected for further analysis. Data from both positive and negative ionization modes were integrated, and the final dataset retained the compounds with the highest identification confidence and lowest CV values, which were compiled into the all_sample_data.xlsx file. Metabolite pathways were annotated using the KEGG database (http://www.kegg.jp) and the Human Metabolome Database (HMDB, https://hmdb.ca/).

### 2.10. Cell Culture

VSMCs were sourced from ATCC and maintained in high‐glucose DMEM medium (12430054, Gibco, United States) enriched with 10% fetal bovine serum (10099141C, Gibco, United States) and 1% penicillin‐streptomycin solution (10378016, Sigma, United States). The culture medium was replaced every 72 h. The cells were then categorized into four distinct experimental groups. Cells in the control group were maintained in standard culture medium without any intervention. Cells in the ox‐LDL group were exposed to 100 *μ*g/mL oxidized low‐density lipoprotein (ox‐LDL, BT‐590, Biomedical Technologies Inc., United States) for a duration of 48 h [27]. Cells in the ox‐LDL + GH group were cotreated with 100 *μ*g/mL ox‐LDL and 100 *μ*g/mL growth hormone (GH) for 48 h [28, 29].

Cells in the ox‐LDL + GH + LY294002 group were exposed to 100 *μ*g/mL ox‐LDL, GH (100 *μ*g/mL), or LY294002 (10 *μ*M, HY10108, MedChemExpress, United States) for 48 h [30].

### 2.11. Fluorescence‐Activated Cell Sorting (FACS) Analysis

Apoptosis and intracellular ROS levels were assessed using FACS (FACSCalibur, BD, United States). Apoptosis was assessed using an apoptosis detection kit (FMSAV647, Fcmacs, China), and ROS levels were determined with an ROS assay kit (S0033S, Beyotime, China), following the manufacturer’s instructions.

### 2.12. Bodipy and Nile Red Staining

To assess lipid accumulation, VSMCs were treated with Bodipy (D3922, Thermo Fisher, United States) or Nile Red (N121291, Aladdin, China) for a period of 20 min. Quantification of the staining intensity was performed using ImageJ software.

## 3. Results

### 3.1. GH Deficiency Promotes the Development of AS in C57BL/6 Mice

**Figure 1:**
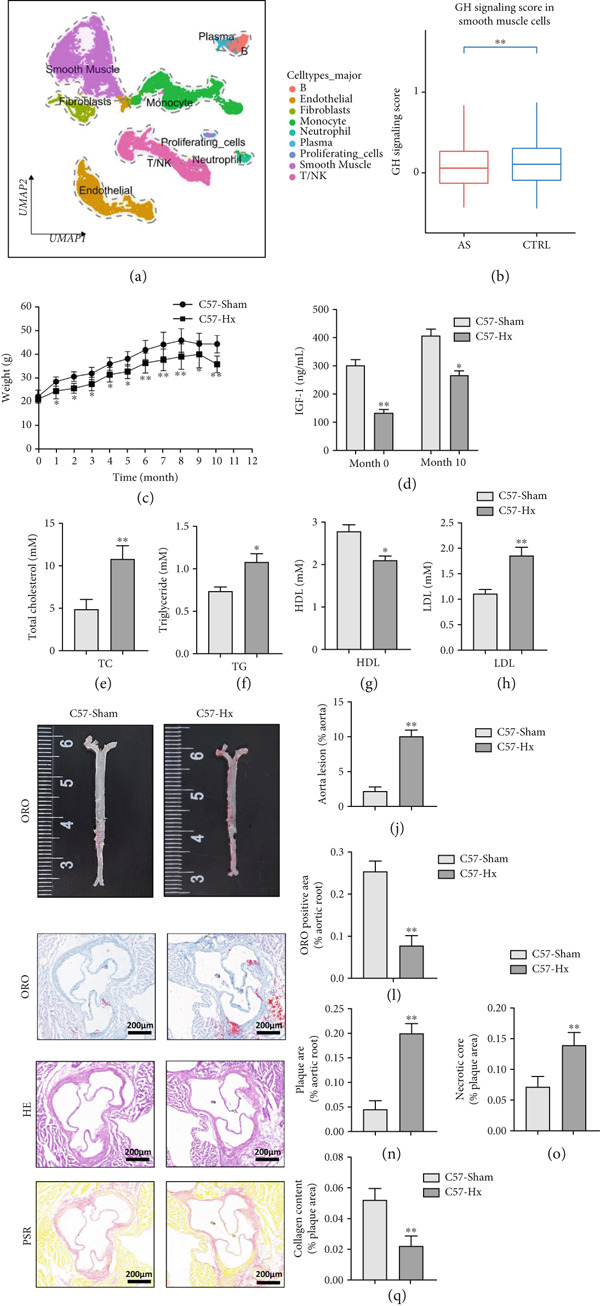
GH deficiency promoted the development of atherosclerosis in C57BL/6 mice. (a) Single‐cell RNA sequencing data of atherosclerotic vascular tissue was downloaded. (b) The expression of GH signal is significantly downregulated in vascular smooth muscle cells. (c) Weight monitoring and statistical comparison of two groups of mice (*n* = 5). (d) Statistical comparison of serum IGF‐1 between the two groups of mice before and after the experiment (*n* = 5). (e–h) Serum contents of TC, TG, HDL, and LDL were detected in biochemical analyzer (*n* = 5). (i, j) The Oil Red O staining of aorta in each group and aortic lesion was quantitatively compared (*n* = 5). (k, l) The arterial lipid stack was evaluated by Oil Red O staining on aortic root sections and Oil Red O–positive area was quantitatively compared (*n* = 5). (m–o) Plaque formation was measured by hematoxylin–eosin staining on aortic root sections; plaque area and necrotic core were quantitatively compared (*n* = 5). (p, q) The collagen content was detected by Picro Sirius Red staining on aortic root sections.  ^∗^ *p* < 0.05 and  ^∗∗^ *p* < 0.01.

scRNA data were obtained from both atherosclerotic and healthy arterial tissues for comprehensive bioinformatics analysis (Figure 1a). We examined the marker genes of various arterial cell types (Figure S1a) and observed that smooth muscle cells constituted a significantly enriched population during the progression of AS (Figure S1b,c). Furthermore, pathway analysis demonstrated that the GH signaling pathway was markedly downregulated in smooth muscle cells isolated from atherosclerotic vascular tissues (Figure 1b), suggesting that GH may play a protective role in the vascular development of AS by regulating the function of VSMCs. To further investigate the effects of GH on AS, the pituitary gland was surgically removed to suppress the GH levels in C57BL/6 mice. Both hypophysectomized and nonhypophysectomized mice were subsequently fed a HFD for 10 months to induce AS. The results demonstrated that HFD markedly increased the body weight of C57‐sham mice over a 10‐month period (Figure 1c). Pituitary removal combined with HFD induced significant hyperlipidemia, characterized by elevated serum TC, TG, and LDL‐C, as well as reduced serum HDL‐C and IGF‐1 in C57‐Hx mice (Figures 1d, 1e, 1f, 1g, and 1h). We further evaluated lesions in the aortic root. HFD led to prominent lipid accumulation in the arteries of C57‐Hx mice (Figure 1i,j), accelerated plaque progression (Figure 1k,l), enhanced necrotic core formation (Figures 1m, 1n, and 1o), and resulted in loss of vascular collagen in C57‐Hx mice (Figure 1p,q). Collectively, these findings indicated that pituitary gland removal elevates blood lipid levels and exacerbates the progression of AS.

### 3.2. GHD Enhances Inflammatory Responses and Oxidative Stress in C57BL/6 Mice

**Figure 2:**
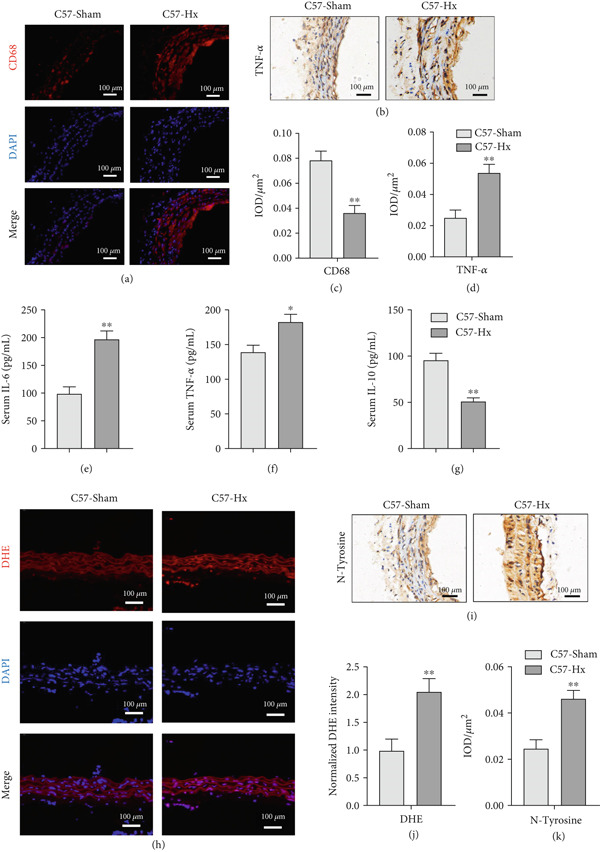
GH deficiency promoted the inflammation and oxidative stress in C57BL/6 mice. (a, c) Immunofluorescence staining and fluorescence intensity quantification comparison of CD68, a macrophage marker in AS plaques (*n* = 5). (b, d) Immunohistochemical staining and quantification comparison of TNF‐*α*, an inflammatory factor in AS plaques (*n* = 5). (e–g) ELISA detection and comparison of serum TNF‐*α*, IL‐6, and IL‐10 levels in the two groups of mice (*n* = 5). (h, j) DHE staining of aortic root sections to measure aortic superoxide and quantitative comparison of fluorescence intensity (*n* = 5). (i, k) Immunohistochemical staining and quantification comparison of N‐tyrosine, an oxidative stress product in AS plaques (*n* = 5).  ^∗^ *p* < 0.05 and  ^∗∗^ *p* < 0.01.

To assess atherosclerotic progression and inflammatory activity, we examined macrophage infiltration in atherosclerotic lesions of C57‐Hx and C57‐sham mice. The plaque area exhibiting CD68‐positive staining, a marker of macrophages, was notably greater in C57‐Hx mice compared to C57‐sham controls (Figure 2a,c). HFD feeding triggered pronounced inflammatory responses in C57‐Hx mice, as evidenced by elevated TNF‐*α* expression and increased serum levels of IL‐6 and TNF‐*α*, along with reduced serum IL‐10 levels (Figures 2b, 2c, 2d, 2e, 2f, and 2g).

To investigate the impact of GH deficiency on superoxide production in the aorta, frozen sections of aortic roots and atherosclerotic plaques were subjected to staining with DHE and anti‐N‐tyrosine antibodies. The results demonstrated that GH deficiency markedly increased superoxide levels (Figure 2h,j) and expression of oxidative stress products, such as N‐tyrosine, in the aortae of C57‐Hx mice (Figure 2i,k).

### 3.3. GH Inhibits Atherosclerotic Progression in Hx ApoE^−/−^ Mice

To further evaluate the effects of GH on AS, we also performed pituitary removal to inhibit GH levels in ApoE^−/−^ mice, which were then subjected to Hx and maintained on a HFD for 12 weeks to promote AS development. The findings demonstrated that administration of GH markedly elevated the body weight of ApoE^−/−^‐Hx mice from Weeks 5–12 (Figure 3a). Pituitary removal combined with HFD induced hyperlipidemia, characterized by elevated serum total TC, TG, and LDL‐C levels, and reduced serum HDL‐C and IGF‐1 levels in ApoE^−/−^‐Hx mice. Administration of GH enhanced the serum lipid profiles in the mouse model (Figures 3b, 3c, 3d, 3e, and 3f).

Figure 3GH suppressed the development of atherosclerosis in ApoE^−/−^ mice with hypophysectomy. (a) Weight monitoring and statistical comparison of four groups of mice (*n* = 5). (b) Statistical comparison of serum IGF‐1 between the four groups of mice before and after the experiment (*n* = 5). (c–f) Serum contents of TC, TG, HDL, and LDL were detected in biochemical analyzer (*n* = 10). (g, h) The Oil Red O staining of aorta in each group and aortic lesion was quantitatively compared (*n* = 5). (i, j) The arterial lipid stack was evaluated by Oil Red O staining on aortic root sections and Oil Red O–positive area was quantitatively compared (*n* = 5). (k–m) Plaque formation was measured by hematoxylin–eosin staining on aortic root sections; plaque area and necrotic core were quantitatively compared (*n* = 5). (n, o) The collagen content was detected by Picro Sirius Red staining on aortic root sections.  ^∗^ *p* < 0.05 and  ^∗∗^ *p* < 0.01.

**Figure:**
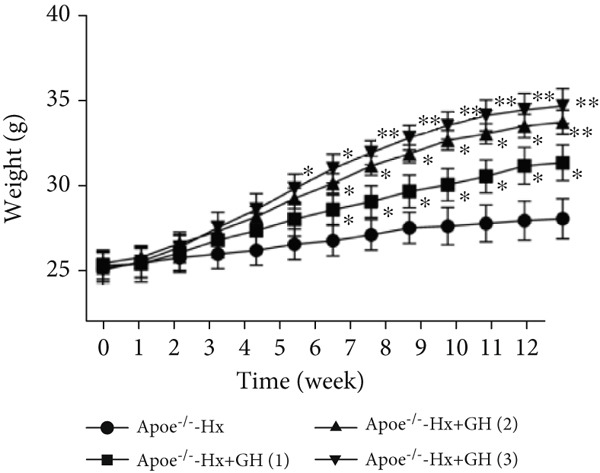
(a)

**Figure:**
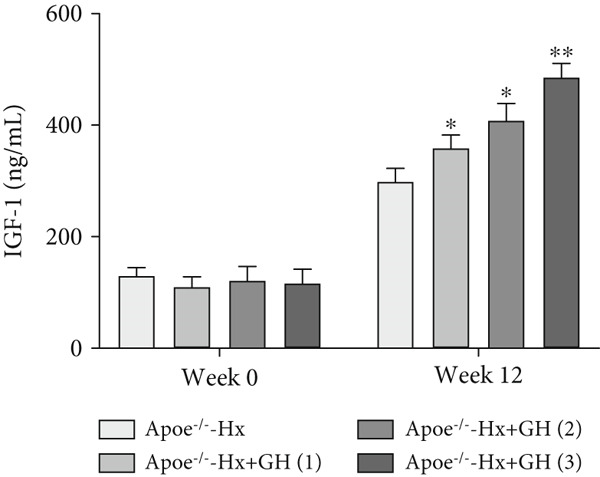
(b)

**Figure:**
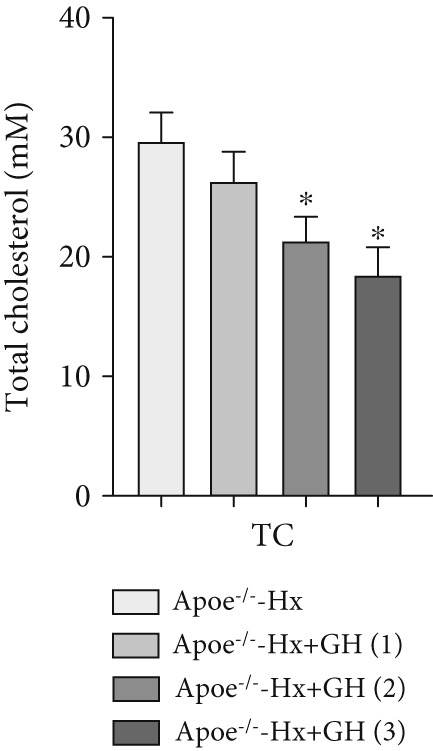
(c)

**Figure:**
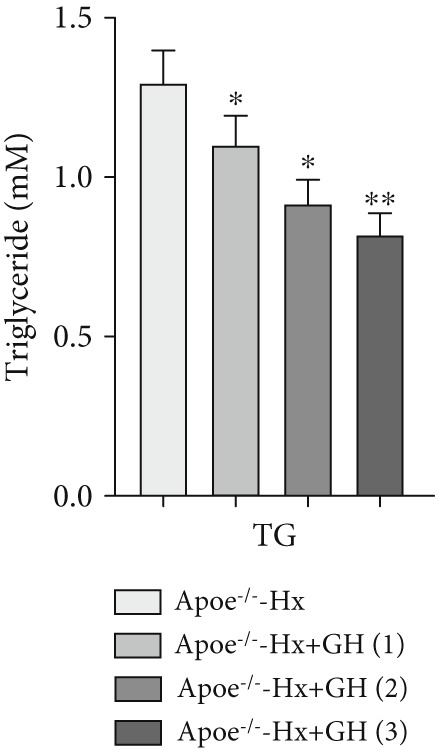
(d)

**Figure:**
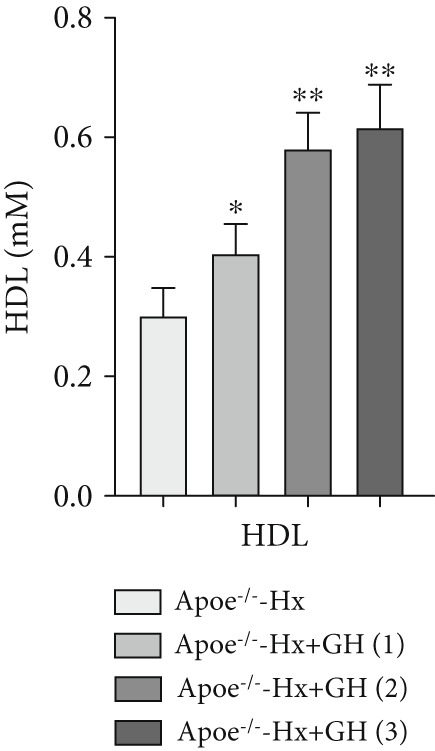
(e)

**Figure:**
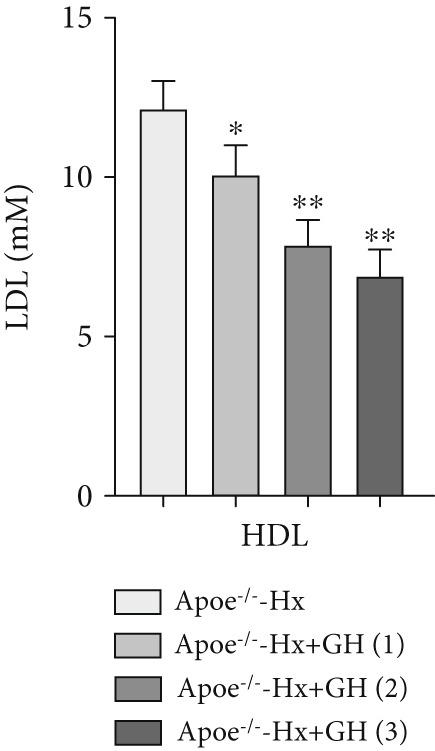
(f)

**Figure:**
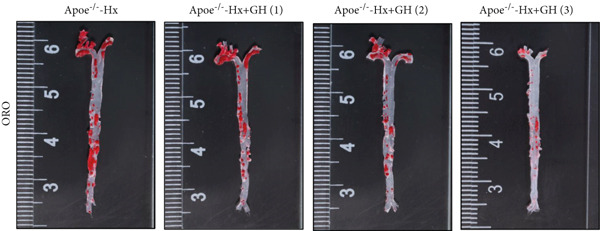
(g)

**Figure:**
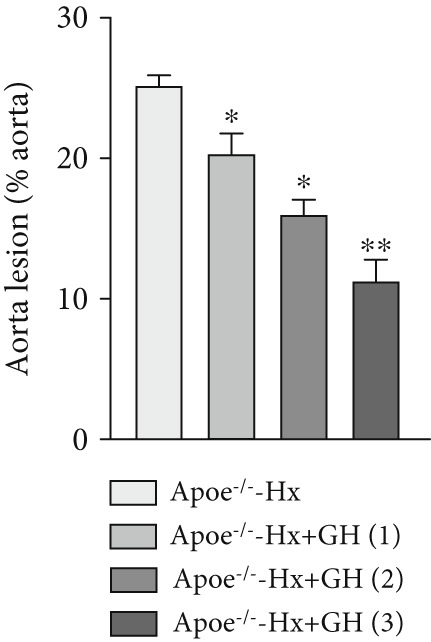
(h)

**Figure:**
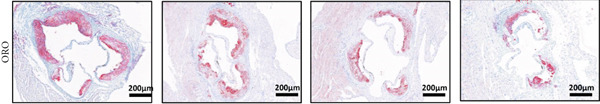
(i)

**Figure:**
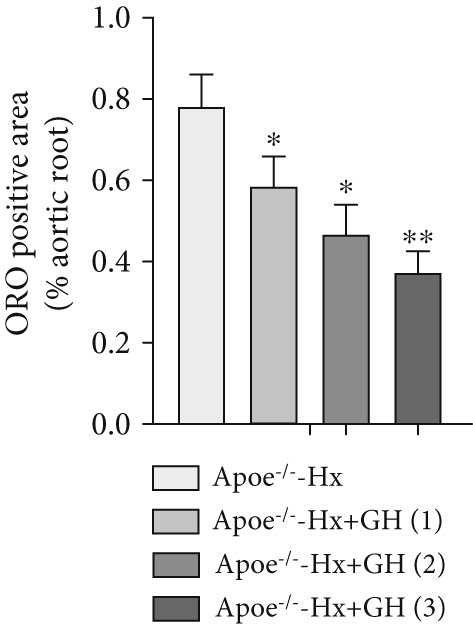
(j)

**Figure:**
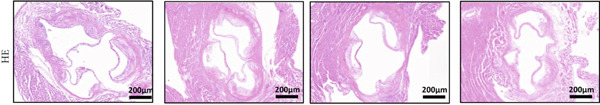
(k)

**Figure:**
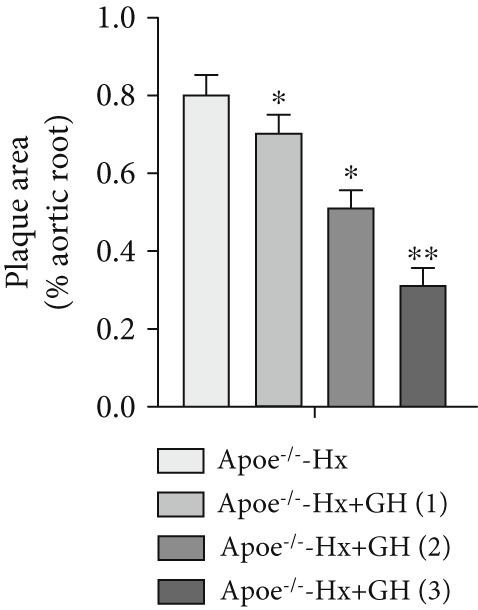
(l)

**Figure:**
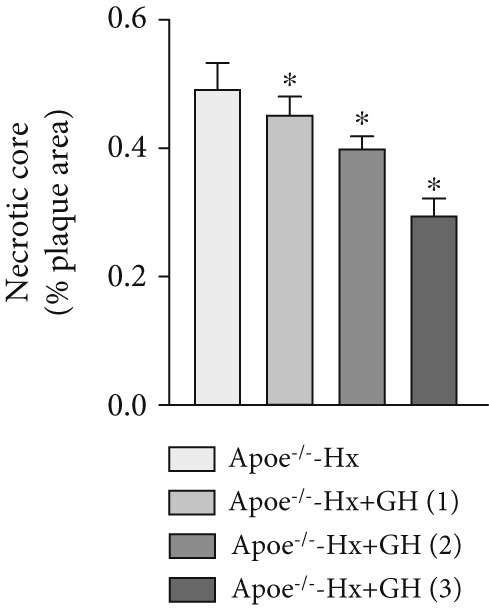
(m)

**Figure:**
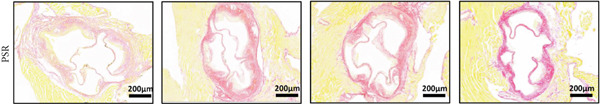
(n)

**Figure:**
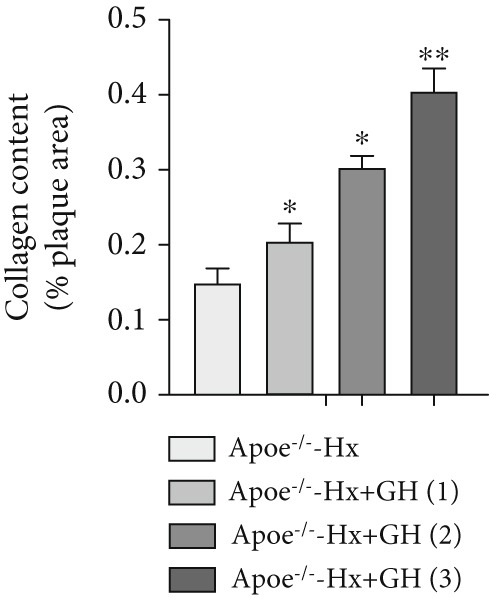
(o)

We conducted further evaluation of lesions in the aortic root. HFD led to substantial lipid deposition in the arteries of ApoE^−/−^‐Hx mice (Figure 3g,h), promoted plaque progression (Figures 3i, 3j, 3k, and 3l), enhanced necrotic core formation (Figure 3k,m), and led to a loss of vascular collagen in ApoE^−/−^‐Hx mice (Figure 3n,o). However, administration of GH suppressed the progression of atherosclerotic plaques in ApoE^−/−^‐Hx mice (Figures 3g, 3h, 3i, 3j, 3k, 3l, 3m, 3n, and 3o). These results suggested that GH contributes significantly to reducing blood lipid levels and inhibiting the development of AS.

### 3.4. GH Suppresses Inflammation in ApoE^−/−^ Hx Mice

We examined the extent of macrophage infiltration and inflammatory activity in atherosclerotic lesions of ApoE^−/−^‐Hx mice. The plaque areas positive for CD68 and TNF‐*α* staining were markedly reduced in ApoE^−/−^‐Hx‐GH mice compared to ApoE^−/−^‐Hx controls (Figures 4a, 4b, 4c, and 4d). Concurrently, HFD feeding triggered significant inflammation, as indicated by elevated serum levels of IL‐6 and TNF‐*α*, along with decreased IL‐10 expression in ApoE^−/−^‐Hx mice (Figures 4e, 4f, and 4g). Administration of GH significantly suppressed serum IL‐6 and TNF‐*α* levels in a dose‐dependent manner while enhancing IL‐10 levels in ApoE^−/−^‐Hx‐GH mice (Figures 4e, 4f, and 4g).

Figure 4GH suppressed the inflammation in ApoE^−/−^‐Hx mice. (a, b) Immunofluorescence staining and fluorescence intensity quantification comparison of CD68, a macrophage marker in AS plaques (*n* = 5). (c, d) Immunohistochemical staining and quantification comparison of TNF‐*α*, an inflammatory factor in AS plaques (*n* = 5). (e–g) ELISA detection and comparison of serum levels of IL‐6, TNF‐*α*, and IL‐10 in the two groups of mice (*n* = 5). Data were presented as mean ± standard deviation in one‐way ANOVA with Dunnett post hoc tests.  ^∗^ *p* < 0.05 and  ^∗∗^ *p* < 0.01.

**Figure:**
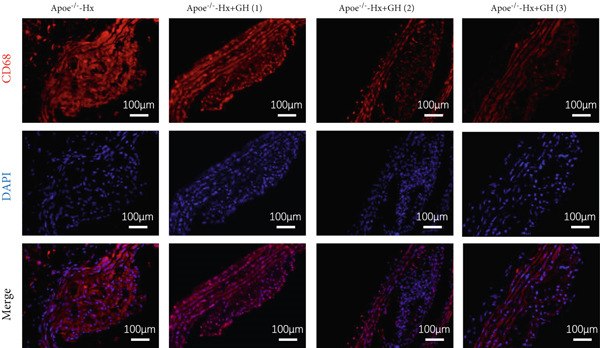
(a)

**Figure:**
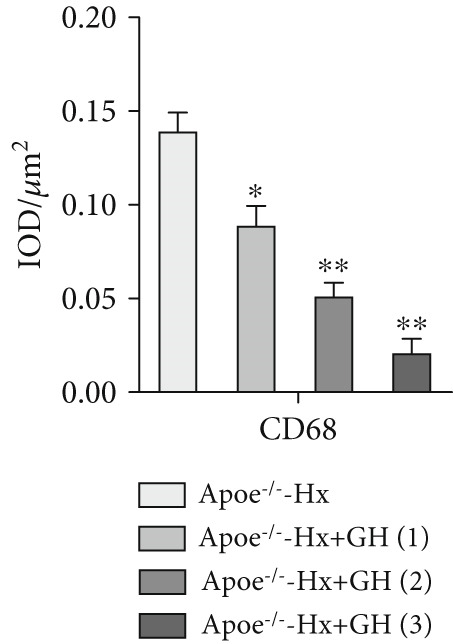
(b)

**Figure:**
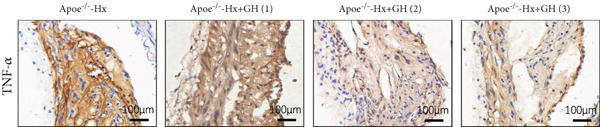
(c)

**Figure:**
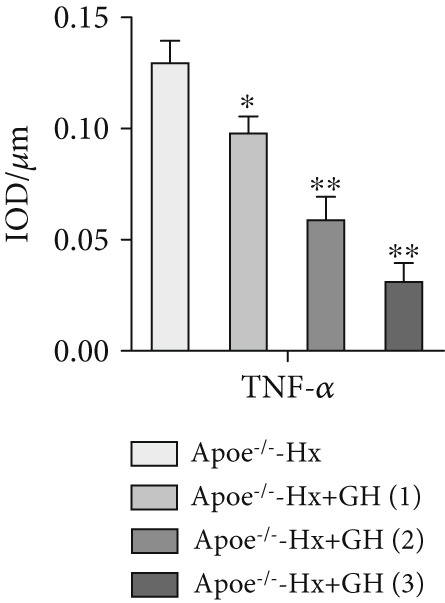
(d)

**Figure:**
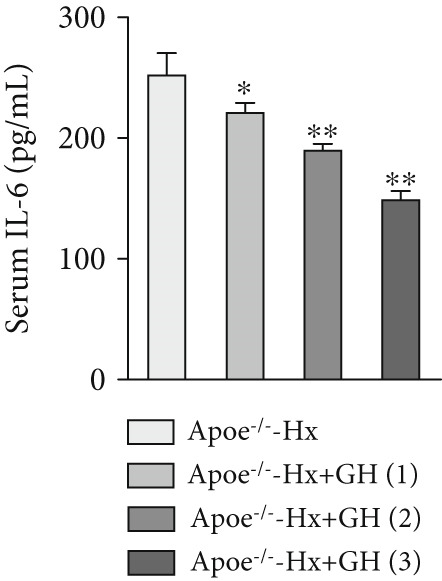
(e)

**Figure:**
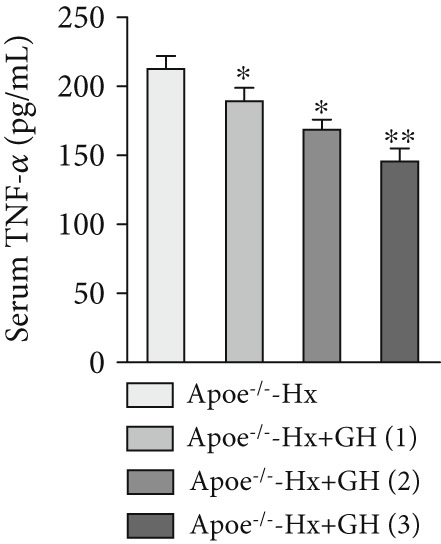
(f)

**Figure:**
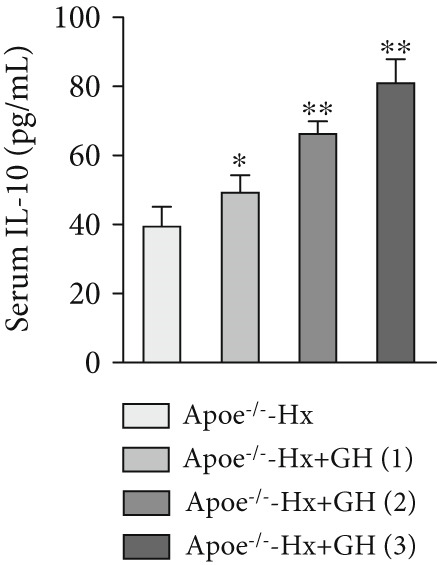
(g)

### 3.5. GH Suppresses ROS Levels and Promotes Apoptosis in the Aorta and AS Plaques of ApoE^−/−^ Hx Mice

To investigate the impact of GH on superoxide production in the aorta, frozen sections of aortic roots and atherosclerotic plaques from ApoE^−/−^‐Hx mice were analyzed using DHE and anti‐N‐tyrosine antibody staining. The results demonstrated that GH significantly decreased superoxide levels in the aorta (Figure 5a,b) and decreased N‐tyrosine levels in the atherosclerotic lesions of GH‐treated ApoE^−/−^‐Hx mice (Figure 5c,d). Additionally, GH treatment promoted apoptosis in atherosclerotic lesions of ApoE^−/−^‐Hx‐GH mice in a dose‐dependent manner (Figure 5e,f).

Figure 5GH suppressed the ROS level and promoted apoptosis in the aorta and AS plaque of ApoE^−/−^‐Hx mice. (a, b) DHE staining of aortic root sections to measure aortic superoxide and quantitative comparison of fluorescence intensity (*n* = 5). (c, d) Immunohistochemical staining and quantification comparison of N‐tyrosine, an oxidative stress product in AS plaques (*n* = 5). (e, f) In situ TUNEL staining of AS plaques to measure apoptosis and quantitative comparison of fluorescence intensity (*n* = 5).  ^∗^ *p* < 0.05 and  ^∗∗^ *p* < 0.01.

**Figure:**
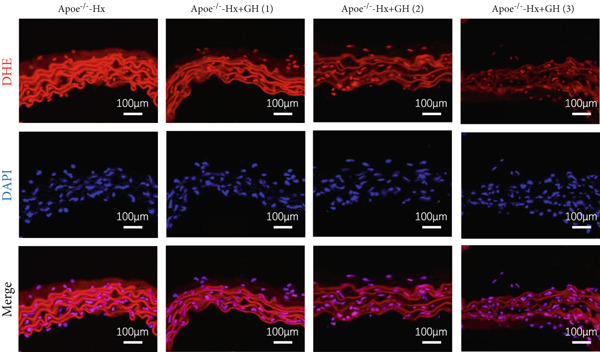
(a)

**Figure:**
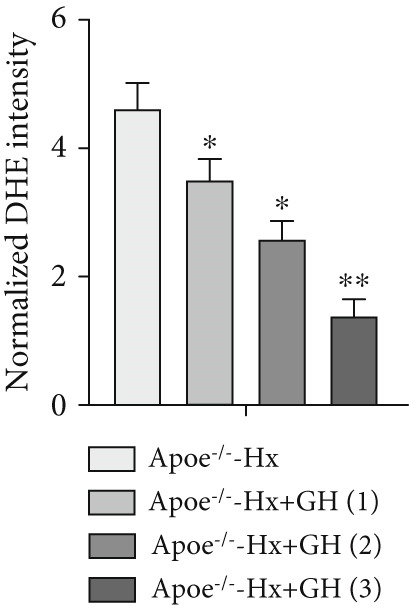
(b)

**Figure:**
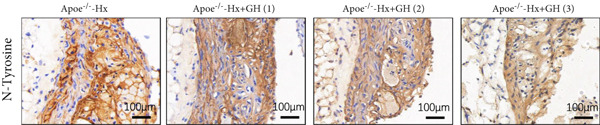
(c)

**Figure:**
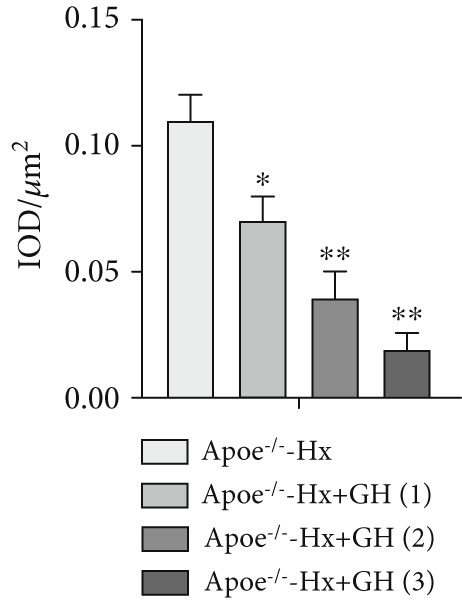
(d)

**Figure:**
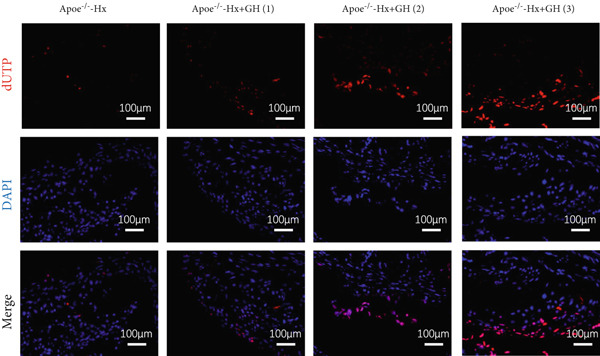
(e)

**Figure:**
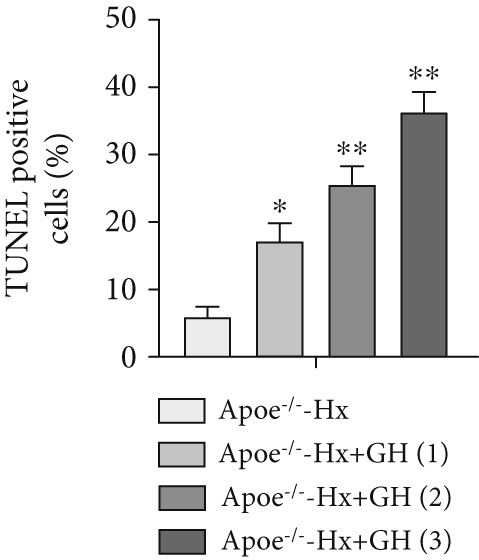
(f)

### 3.6. GH Changed Metabolites and Metabolic Pathways in ApoE^−/−^‐Hx Mice

To assess the impact of GH on metabolic profiles and associated pathways, serum samples from C57‐sham, C57‐Hx, ApoE^−/−^‐Hx, and ApoE^−/−^‐Hx‐GH(3) mice were analyzed by LC‐MS. The heatmap revealed that GH induced differential lipid metabolism in the mice (Figure S2a,b). A total of 1404 significantly altered metabolites were detected when comparing C57‐sham and C57‐Hx mice, while 1045 differential metabolites were observed between ApoE^−/−^‐Hx and ApoE^−/−^‐Hx‐GH(3) mice. Specifically, in comparison to the GH‐sham group, 33 metabolites showed increased levels and 202 were decreased in the GH‐Hx group (Figure S2c). Additionally, when compared to the ApoE^−/−^‐Hx + GH(3) group, 180 metabolites were upregulated and 415 were downregulated in the ApoE^−/−^‐Hx group (Figure S2d).

The enriched metabolic pathways involved in these differential metabolites are shown in Figure 6. Topological analysis of metabolic pathways in C57‐Hx and C57‐sham mice revealed alterations predominantly in pathways related to fatty acid metabolism, elongation, degradation, biosynthesis, and the synthesis of unsaturated fatty acids (Figure 6a). Differences in the metabolic profiles between ApoE^−/−^‐Hx and ApoE^−/−^‐Hx‐GH(3) mice were mainly attributed to disruptions in pathways involving fatty acid metabolism, elongation, amino acid synthesis, and the production of unsaturated fatty acids (Figure 6b). These pathways are closely associated with lipid metabolism. Furthermore, analysis of serum metabolite composition showed that compared with the C57‐sham and ApoE^−/−^‐Hx‐GH(3) groups, GH deficiency in the C57‐sham and ApoE^−/−^‐Hx + GH(3) groups resulted in increased levels of three metabolites (POPC, DMPE, and 4‐chloro‐2‐nitrobenzylalcohol) and a decrease in one metabolite (1‐heptadecanoyl‐sn‐glycerol‐3‐phosphocholine) (Figure 6c,d). Notably, POPC, DMPE, and 1‐Heptadecanoyl‐sn‐glycerol‐3‐phosphocholine have been reported to exert regulatory effects on lipid metabolism [31–33]. These phospholipids play crucial roles in maintaining cellular functions and influencing metabolic pathways [33]. The above results indicated that the metabolic characteristics and changes in the C57‐sham and C57‐Hx groups were similar to those in the ApoE^−/−^‐Hx and ApoE^−/−^‐Hx‐GH(3) groups after pituitary removal and GH intervention, suggesting that GH has a significant impact on lipid metabolism in mice. These results underscore the critical function of GH in maintaining lipid homeostasis through the upregulation of POPC, DMPE, and 4‐chloro‐2‐nitrobenzylalcohol, as well as the downregulation of 1‐heptadecanoyl‐sn‐glycerol‐3‐phosphocholine in mice.

Figure 6GH changed metabolites and metabolic pathways in ApoE^−/−^‐Hx mice (a) The topological analysis of the metabolic pathways between C57‐Hx and C57‐sham mice (*n* = 5). (b) The topological analysis of the metabolic pathways between ApoE^−/−^‐Hx and ApoE^−/−^‐Hx‐GH(3) mice (*n* = 5). (c) The analysis of serum metabolite composition between C57‐Hx and C57‐sham mice (*n* = 5). (d) The analysis of serum metabolite composition between ApoE^−/−^‐Hx and ApoE^−/−^‐Hx‐GH(3) mice (*n* = 5). Data were presented as mean ± standard deviation in one‐way ANOVA with Dunnett post hoc tests.  ^∗^ *p* < 0.05 and  ^∗∗^ *p* < 0.01.

**Figure:**
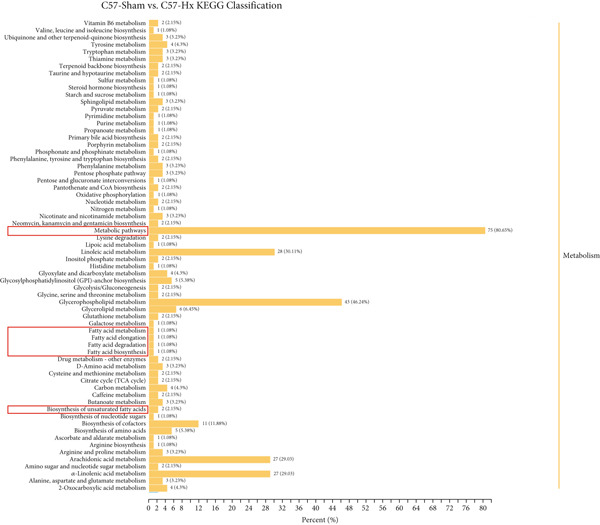
(a)

**Figure:**
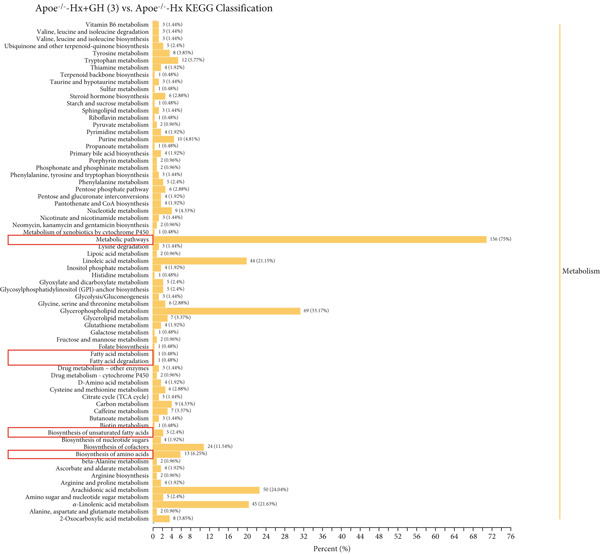
(b)

**Figure:**
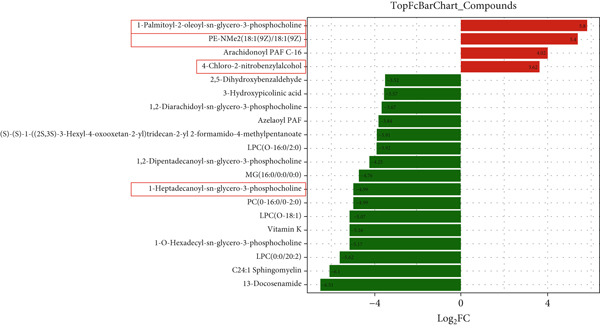
(c)

**Figure:**
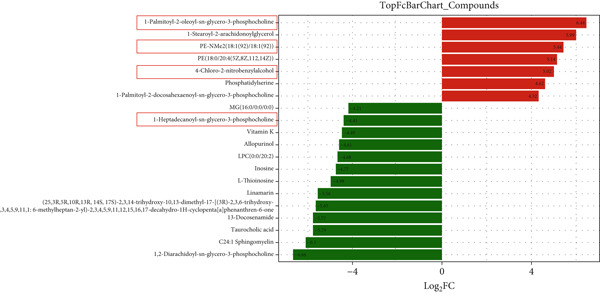
(d)

### 3.7. GH Demonstrates a Protective Role in Atherosclerotic Vascular Tissues via the PI3K/AKT Signaling Pathway

RNA sequencing revealed significant differences in gene expression within the aortic vessels of C57‐sham and C57‐Hx mice (Figure S3a). Specifically, in C57‐Hx mice, 1940 genes showed increased expression, while 1955 genes exhibited reduced expression compared to C57‐sham mice (Figure S3b). This widespread variation in gene expression indicates that the aortic tissues from the two groups are involved in different biological activities. To gain deeper insight into the molecular mechanisms driving these changes, we conducted a comprehensive pathway analysis of the differentially expressed genes. The analysis highlighted fatty acid oxidation and oxidative phosphorylation as key pathways involved (Figure 7a). These pathways are crucial for cellular energy production and metabolic regulation, indicating that changes in these processes may contribute to the observed differences between the C57‐sham and C57‐Hx groups. Furthermore, KEGG pathway analysis provided additional insights, indicating that the PI3K/AKT signaling pathway may serve as a key mediator of these biological effects (Figure 7b).

Figure 7GH exerts a protective effect on atherosclerotic vascular tissues through the PI3K/AKT signaling pathway. (a) Statistical analysis of the pathways associated with differentially expressed genes. (b) KEGG enrichment analysis of differentially expressed genes. (c) Western blotting on the expression of p‐PI3K, p‐AKT, IL‐18, 1L‐1*β*, and cleaved caspase 1 in the C57‐Hx and C57‐sham groups. (d) Western blotting on the expressions of p‐PI3K, p‐AKT, IL‐18, 1L‐1*β*, and cleaved caspase 1. Compared with the control group,  ^∗^ *p* < 0.05 and  ^∗∗^ *p* < 0.01. Compared with the ApoE^−/−^‐Hx group, ^**#**^ *p* < 0.05 and ^**##**^ *p* < 0.01.

**Figure:**
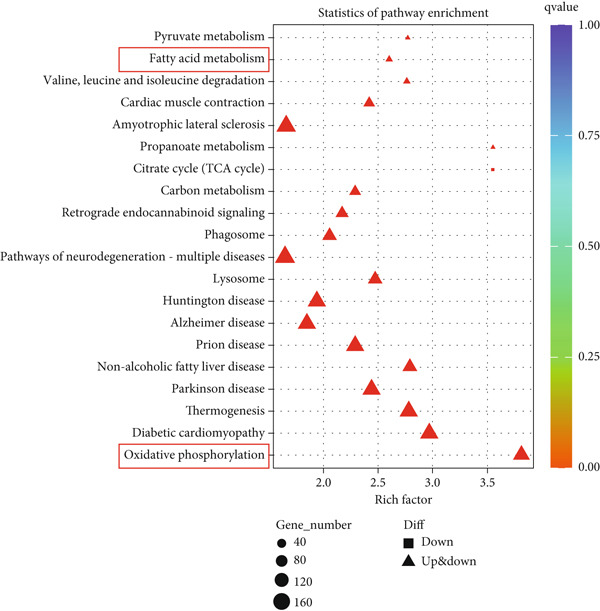
(a)

**Figure:**
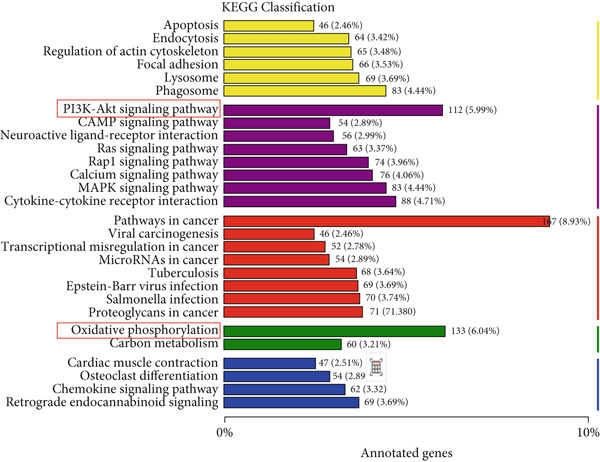
(b)

**Figure:**
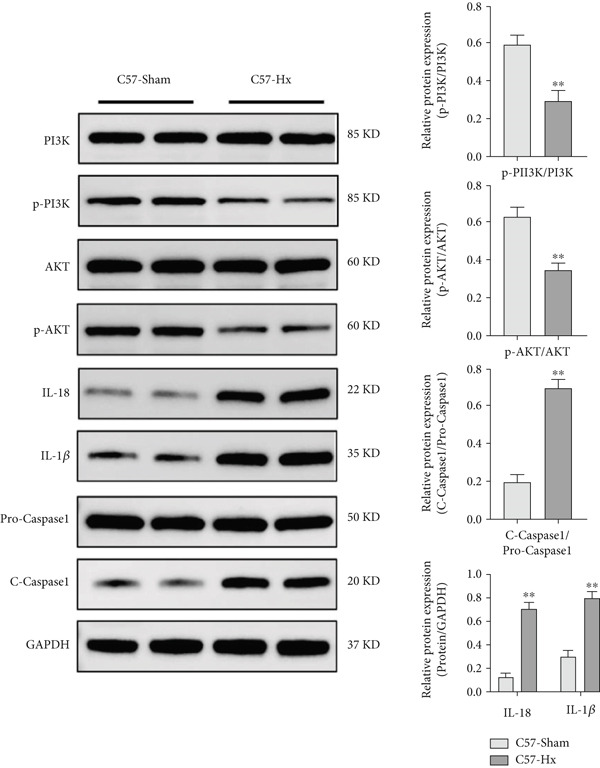
(c)

**Figure:**
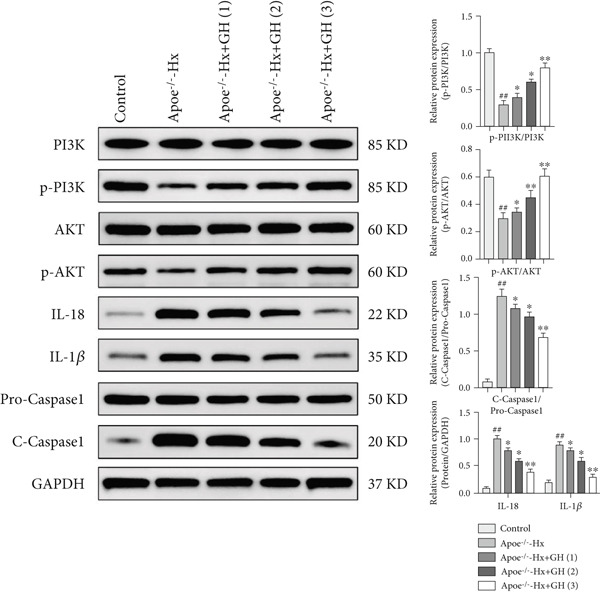
(d)

The PI3K/AKT pathway is widely recognized for its role in regulating cell survival, growth, and metabolic processes [34, 35], which positions it as a strong candidate for further exploration. Our findings revealed that the phosphorylated forms of PI3K and AKT (p‐PI3K and p‐AKT) were notably decreased in the aortic tissues of C57‐Hx mice when compared to the control group (Figure 7c). Alongside these changes in the PI3K/AKT signaling cascade, we also detected significant modifications in the expression of inflammatory mediators and apoptosis‐related proteins. In particular, the levels of IL‐18 and IL‐1*β*, both key proinflammatory cytokines, were substantially increased in the C57‐Hx group, and the expression of cleaved caspase 1, an indicator of apoptotic activation, was also elevated (Figure 7c).

To explore potential therapeutic interventions, we examined the effects of GH on aortic‐related proteins in several mouse models, including ApoE^−/−^‐Hx, ApoE^−/−^‐Hx‐GH(1), ApoE^−/−^‐Hx‐GH(2), and ApoE^−/−^‐Hx‐GH(3). Normal C57BL/6 mice were used as controls. Our data revealed that the administration of GH led to a dose‐dependent increase in the phosphorylation of PI3K and AKT, consequently boosting the activation of the PI3K/AKT signaling cascade (Figure 7d). Furthermore, GH treatment significantly reduced the expression of the proinflammatory cytokines IL‐18 and IL‐1*β*, along with the apoptotic marker cleaved caspase 1 (Figure 7d). Taken together, these results suggested that GH exerts its protective effects against AS by stimulating the PI3K/AKT pathway, which in turn supports cell survival while attenuating inflammatory responses and programmed cell death.

### 3.8. GH Demonstrates a Protective Role in ox‐LDL‐Induced VSMCs via Activation of the PI3K/AKT Signaling Pathway

**Figure 8:**
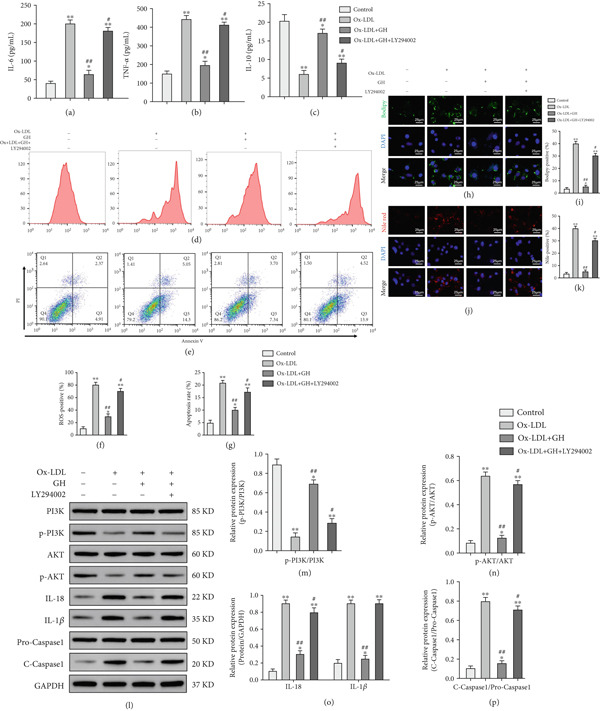
GH exerts a protective effect on VSMC cells induced by ox‐LDL through the PI3K/AKT signaling pathway. (a–c) Serum contents of IL‐6, TNF‐*α*, and IL‐10 were detected by ELISA (*n* = 5). (d, f) ROS levels in VSMC cells were detected using flow cytometry (*n* = 5). (e, g) Apoptosis was detected using flow cytometry (*n* = 5). (h–k) Lipid deposition was detected using Bodipy and Nile red staining. (l–p) Western blotting on the expression of p‐PI3K, p‐AKT, IL‐18, 1L‐1*β*, and cleaved caspase 1 in VSMC cells. Compared with the control group,  ^∗^ *p* < 0.05 and  ^∗∗^ *p* < 0.01. Compared with the ApoE^−/−^‐Hx group, ^**#**^ *p* < 0.05 and ^##^ *p* < 0.01.

Considering the pivotal involvement of VSMCs in the development of AS [36–38], it is crucial to further explore the effects and molecular mechanisms of GH in vascular AS. To this end, we conducted an experiment where we induced an atherosclerotic phenotype in mouse VSMCs using ox‐LDL. The cells were then cocultured with GH and/or the PI3K pathway inhibitor LY294002. Our study had several important findings. First, ox‐LDL enhanced the levels of foam cell–specific markers, IL‐6 and TNF‐*α*, while downregulating IL‐10 content in VSMCs (Figures 8a, 8b, and 8c). GH inhibited these changes, whereas LY294002 counteracted the protective effects of GH. Flow cytometry analysis demonstrated that ox‐LDL significantly increased ROS levels and apoptosis in VSMCs (Figures 8d, 8e, 8f, and 8g). However, when GH was introduced, these adverse effects were significantly inhibited. Interestingly, LY294002 reversed the protective effects of GH, indicating that the PI3K pathway is a key mediator of the positive effects of GH.

Furthermore, the internalization of lipid droplets by VSMCs represents a critical step in the development of foam cells [27]. Bodipy and Nile Red fluorescence staining provided additional insights into lipid accumulation in VSMCs. Exposure to ox‐LDL resulted in a significant enhancement of lipid deposition, which was effectively suppressed by GH (Figures 8h, 8i, 8j, and 8k). LY294002 abolished the suppressive effect of GH on lipid buildup, further highlighting the essential role of the PI3K pathway in this mechanism.

The Western blot analysis provided additional confirmation of the engagement of the PI3K/AKT signaling pathway (Figures 8l, 8m, 8n, 8o, and 8p). ox‐LDL exposure resulted in decreased phosphorylation status of PI3K and AKT in VSMCs, while simultaneously increasing the production of proinflammatory cytokines, including IL‐18 and IL‐1*β*, as well as the apoptotic protein cleaved caspase 1 (Figures 8l, 8m, 8n, 8o, and 8p). GH significantly inhibited these changes, whereas LY294002 counteracted the protective effect of GH (Figures 8l, 8m, 8n, 8o, and 8p). Collectively, these results indicated that GH protects VSMCs by suppressing the progression of AS via activation of the PI3K/AKT signaling pathway.

## 4. Discussion

GH has significant implications in cardiovascular physiology that extend beyond its established roles in growth and metabolic regulation. It influences cardiac architecture and contractile function [39], and both experimental and clinical GHD are linked to heightened systemic vascular resistance [40], diminished vascular responsiveness [41], and a lipid profile resembling that observed in AS‐related dyslipidemia [42]. These alterations may underlie the elevated risk of cardiovascular mortality seen in individuals with GHD [43]. Moreover, the incidence of hypertension [39] and AS [44] is notably greater in GHD patients, and those with hypopituitarism exhibit a markedly increased cardiovascular morbidity and mortality [45]. GH replacement therapy in GHD has been demonstrated to normalize vascular resistance [40], reduce arterial stiffness [46], enhance vasodilation [41], and reverse early markers of AS [44]. Recombinant GH treatment can correct lipid disturbances, lower AS indices, decrease cardiovascular risk, reduce mortality, and improve quality of life [47]. Preclinical evidence also suggests that GHD accelerates AS progression [19]. However, GH administration in non‐GHD populations is associated with several risks, including insulin resistance, dyslipidemia, cardiomyopathy, hypertension, and potentially tumor development due to GH/IGF‐1 signaling. In children without GHD, GH therapy may lead to gigantism [48]. Therefore, GH should not be used as a routine intervention unless clearly indicated by established clinical guidelines [48, 49].

In this study, scRNA data indicated that GH might exert inhibitory effects on the vascular development of AS through modulation of VSMCs’ activity. To elucidate the role and underlying mechanisms of GH in AS, this study investigates GH function using well‐established Hx C57BL/6 and ApoE^−/−^ mouse models [9–11]. Hx mice exhibited marked aortic plaque formation under HFD, along with increased serum lipids, oxidative stress, and inflammatory responses. Administration of GH significantly attenuated plaque development, inflammation, and oxidative stress in ApoE^−/−^ mice, demonstrating its protective effects against AS. Serum lipid profiling demonstrated that GH modulates lipid metabolism by altering lipoprotein and cholesterol levels. Elevated GH levels correlated with increased concentrations of POPC, DMPE, and 4‐chloro‐2‐nitrobenzyl alcohol, whereas 1‐heptadecanoyl‐sn‐glycerol‐3‐phosphocholine was significantly reduced. POPC and DMPE are glycerophospholipids involved in cell membrane structure and function [31]. 1‐Heptadecanoyl‐sn‐glycerol‐3‐phosphocholine, a lysophosphatidylcholine (LPC) derivative, promotes oxidative stress, inflammation, and early atherosclerotic lesion formation and impairs vascular function by inhibiting nitric oxide synthesis [32]. Collectively, these results underscore the critical importance of GH in maintaining lipid homeostasis through the down‐regulation of 1‐heptadecanoyl‐sn‐glycerol‐3‐phosphocholine, a compound critical to cardiovascular health.

VSMCs play a critical role in AS by transforming into foam cells, which drive inflammation and destabilize atherosclerotic plaques [36, 50]. The viability of VSMCs directly influences the structural integrity of the fibrous cap, and their apoptosis contributes to disease progression [51–53]. Modulating the formation of foam cells derived from VSMCs presents a potential therapeutic approach for AS. Cellular mechanisms associated with AS were examined using in vitro experimental models. VSMCs were exposed to ox‐LDL, a well‐established inducer of oxidative stress and apoptosis that mimics pathological conditions during AS [54]. GH treatment significantly reduced ox‐LDL‐induced oxidative stress and apoptosis, while also suppressing the generation of proinflammatory cytokines in VSMCs. Furthermore, GH inhibited lipid accumulation, another key contributor to atherosclerotic lesion formation. Importantly, the positive effects of GH were negated by the PI3K/AKT pathway inhibitor LY294002, confirming that its protective role is mediated through this signaling cascade.

Consistent with the scRNA‐seq analysis, our findings demonstrated that GH mitigated the progression of AS by modulating the PI3K/AKT pathway, a signaling cascade critically involved in inflammatory regulation and apoptosis during plaque development [55]. Activation of this pathway decreases ROS levels and lipid accumulation, thereby suppressing plaque formation and attenuating disease progression [56]. Gu et al. demonstrated that enhancing PI3K/AKT signaling protects endothelial cells, alleviates oxidative stress, and inhibits AS in ApoE^−/−^ mice [57]. AKT directly inactivates BAX, a proapoptotic protein, which relieves its suppression of BCL‐2, thereby reducing apoptosis and promoting cell survival [58]. Accumulation of ROS compromises the antioxidant defense system and impairs DNA synthesis, ultimately exacerbating inflammatory responses [59]. Therefore, enhancing PI3K/AKT pathway activity may serve as a promising therapeutic approach for reducing inflammation and apoptosis associated with atherosclerotic development [60].

Research has demonstrated a link between the GH/IGF‐I axis and cardiovascular well‐being by identifying both functional and structural impairments in patients with abnormal GH regulation [47]. According to a study by Sherlock et al., individuals with hypopituitarism have a shorter life span and face approximately double the risk of cardiovascular death when compared to healthy individuals [61]. Moreover, GHD may be associated with elevated mortality rates, even among patients receiving replacement therapy for other pituitary hormones [23]. IGF‐1 enhances cardiovascular function, reduces cardiovascular mortality [62], and exerts protective effects against AS [63]. While GH stimulates IGF‐I expression across multiple tissues [64], it does not appear to regulate IGF‐I transcription in vascular endothelial or smooth muscle cells [65]. Our findings demonstrate that GH can independently confer protection against AS through stimulation of the PI3K/AKT signaling cascade. Recent studies also highlight the protective effects of GH on ovarian function in sheep [29], which are achieved by reducing oxidative stress and inhibiting apoptosis in granulosa cells through the aforementioned pathway.

In summary, the present study reveals that GH exerts an inhibitory effect on the development of AS through activation of the PI3K/AKT signaling pathway in VSMCs. These results highlight the therapeutic promise of GH in managing AS and suggest that the PI3K/AKT pathway could serve as a valuable target for future clinical treatments.

## Ethics Statement

All procedures involving animals were authorized by the Institutional Animal Care and Use Committee (IACUC) at Nanjing Medical University, and the experimental methods were conducted following the guidelines outlined in the approved protocol.

## Consent

The authors have nothing to report.

## Disclosure

All authors approved the final version for publication.

## Conflicts of Interest

The authors declare no conflicts of interest.

## Author Contributions

D.H. and B.L. were responsible for the conception and design of the study. Yu.W. and X.Z. carried out material preparation, while J.C., S.S., and X.W. were involved in data collection. Data analysis was conducted by Ya.W. and Y.S. D.H. and B.L. drafted the initial version of the manuscript. All authors contributed to critical revisions of the manuscript for important intellectual content. J.C. and S.S. contributed equally.

## Funding

This work was supported by the Scientific Research Project of Nantong Municipal Health Commission (MS2024012).

## Supporting information


**Supporting Information** Additional supporting information can be found online in the Supporting Information section. Figure S1: Comparative analysis of cell type composition in the aortas of atherosclerotic and control mice based on scRNA‐seq data. (a) Determination of marker genes for distinct cell populations in the mouse aorta for single‐cell analysis. (b, c) Comparative cell type profiling of aortas from atherosclerotic and control mice. Figure S2: GH induced differential lipid metabolism in mice. (a) Heatmap of serum metabolites in hypophysectomized mice and control mice detected by LC‐MS (*n* = 5). (b) Heatmap of serum metabolites in serum of GH‐treated ApoE^−/−^‐Hx mice and control ApoE^−/−^‐Hx mice detected by LC‐MS (*n* = 5). (c) Volcano plot of differential metabolites in hypophysectomized mice and control mice (*n* = 5). (d) Volcano plot of differential metabolites in serum of GH‐treated ApoE^−/−^‐Hx mice and control ApoE^−/−^‐Hx mice (*n* = 5). Figure S3: Pituitary removal caused differential expression of aortic vascular genes. (a) Gene expression heatmap of abdominal aorta and control vessels in pituitary depleted mice detected by RNA sequencing (*n* = 3). (b) Volcano plot of differentially expressed genes in the abdominal aorta and control vessels of pituitary depleted mice (*n* = 3).

## Data Availability

The data supporting the findings of this study can be obtained from the corresponding author upon reasonable request.
