# Supplementary material for: Growth Hormone Alleviates Atherosclerosis Through Regulating the Activity of PI3K/AKT Pathway: Insights From Single‐Cell Sequence and Mechanism Exploration
Source: Int J Genomics. 2025 Oct 13;2025:9710652. doi: 10.1155/ijog/9710652 (PMC12516355; doi:10.1155/ijog/9710652)

**Supplementary files for**

**Growth hormone alleviates atherosclerosis through regulating the activity of PI3K/AKT pathway：insights from single cell sequence and mechanism exploration**

Jin Cai^1^*, Shuang Shi^2^*, Yuhang Wang^2^, Xiangdong zhang^1^, Xinghua Wei^2^, Yanjing Wu^2^, Yunlong Shi^2^, Bin Li^1^, Daorong Hou^3^

1 Department of Pediatrics, Affiliated Hospital of Nantong University, No. 19 Qixiu Road, Chongchuan District, Nantong 226001, China

2 Department of Pediatrics, Medical College of Nantong University, No. 688 Qingnian East Road, Nantong 226001, China

3 Key Laboratory of Model Animal Research, Animal Core Facility, Nanjing Medical University, No. 101 Longmian Avenue, Jiangning District, Nanjing 211166, China

**Supplementary methods**

**Transcriptomic Analysis of Aorta**

Total RNA was extracted from mouse abdominal aortic tissues using TRIzol reagent (B5704-1, Takara, Japan) following the manufacturer's protocol. RNA quantity and integrity were evaluated using an Agilent 2100 Bioanalyzer (Agilent Technologies, USA), and purity was further confirmed with a NanoDrop spectrophotometer (IMPLEN, USA). RNA sequencing (RNA-seq) was conducted on an Illumina second-generation sequencing platform (PE150 read length) at Tsingke Biology (China). Differential gene expression between the two experimental groups was analyzed using the DESeq R package (version 1.10.1). Genes exhibiting a |log2 fold change| > 1 and a P-value < 0.05 were considered differentially expressed. Functional enrichment analysis, including Gene Ontology (GO) and KEGG pathway annotations, was carried out using the GOseq R package and KOBAS software, respectively.

**Western Blot Assays**

Protein content in vascular tissue and VSMCs was determined using the BCA Protein Assay Kit (P0009, Beyotime, China). A total of 30 µg of protein lysate was subjected to SDS-PAGE separation and subsequently transferred onto nitrocellulose membranes (HATF00010, Millipore, USA) via wet transfer. The membranes were incubated with primary antibodies targeting phosphorylated (p)-PI3K (P85 Tyr458) (17366, 1:1000, CST, USA), PI3K (4257, 1:1000, CST, USA), p-AKT (Ser473) (4060, 1:1000, CST, USA), AKT (4685, 1:1000, CST, USA), IL-1β (GB122059, 1:1000, Servicebio), Caspase-1 (A16792, 1:1000, ABclonal), IL-18 (A1115, 1:500, ABclonal, China), and GAPDH (GB15002, Servicebio). GAPDH (ab9482, 1:1000, Abcam, USA) served as the internal control for signal normalization. Densitometric analysis of the protein bands was performed using ImageJ software.

**Statistical Analysis**

Results are expressed as mean ± standard deviation (SD). For comparisons across multiple groups, one-way analysis of variance (ANOVA) was conducted, followed by Tukey's post hoc test. When comparing two groups, an unpaired Student's t-test was applied. A p-value less than 0.05 was considered statistically significant. All statistical analyses were performed using GraphPad Prism software (version 10.2.0).

**Supplementary figures**

**Fig. S1 Comparative analysis of cell type composition in the aortas of atherosclerotic and control mice based on scRNA-seq data.**

(A) Determination of marker genes for distinct cell populations in the mouse aorta for single-cell analysis. (B, C) Comparative cell type profiling of aortas from atherosclerotic and control mice.


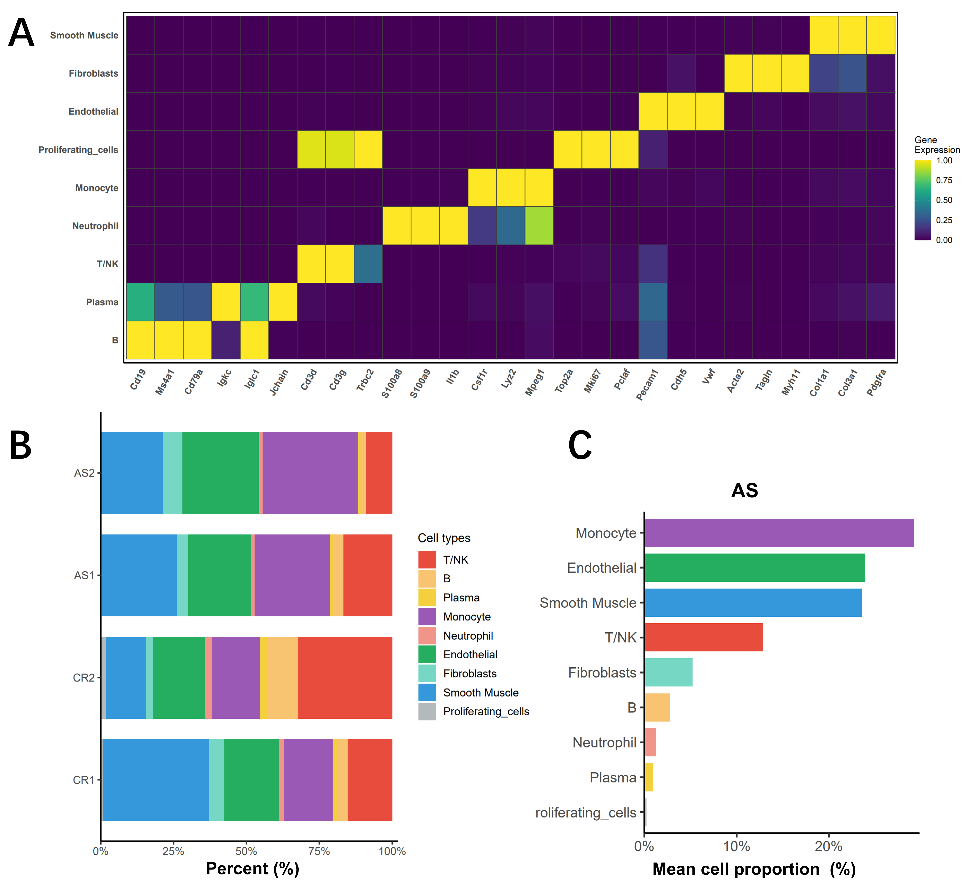


**Fig. S2 GH induced differential lipid metabolism in mice.**

(A) Heatmap of serum metabolites in hypophysectomized mice and control mice detected by LC-MS (n=5). (B) Heatmap of serum metabolites in serum of GH-treated ApoE^-/-^-Hx mice and control ApoE^-/-^-Hx mice detected by LC-MS (n=5). (C) Volcano plot of differential metabolites in hypophysectomized mice and control mice (n=5). (D) Volcano plot of differential metabolites in serum of GH-treated ApoE^-/-^-Hx mice and control ApoE^-/-^-Hx mice (n=5).


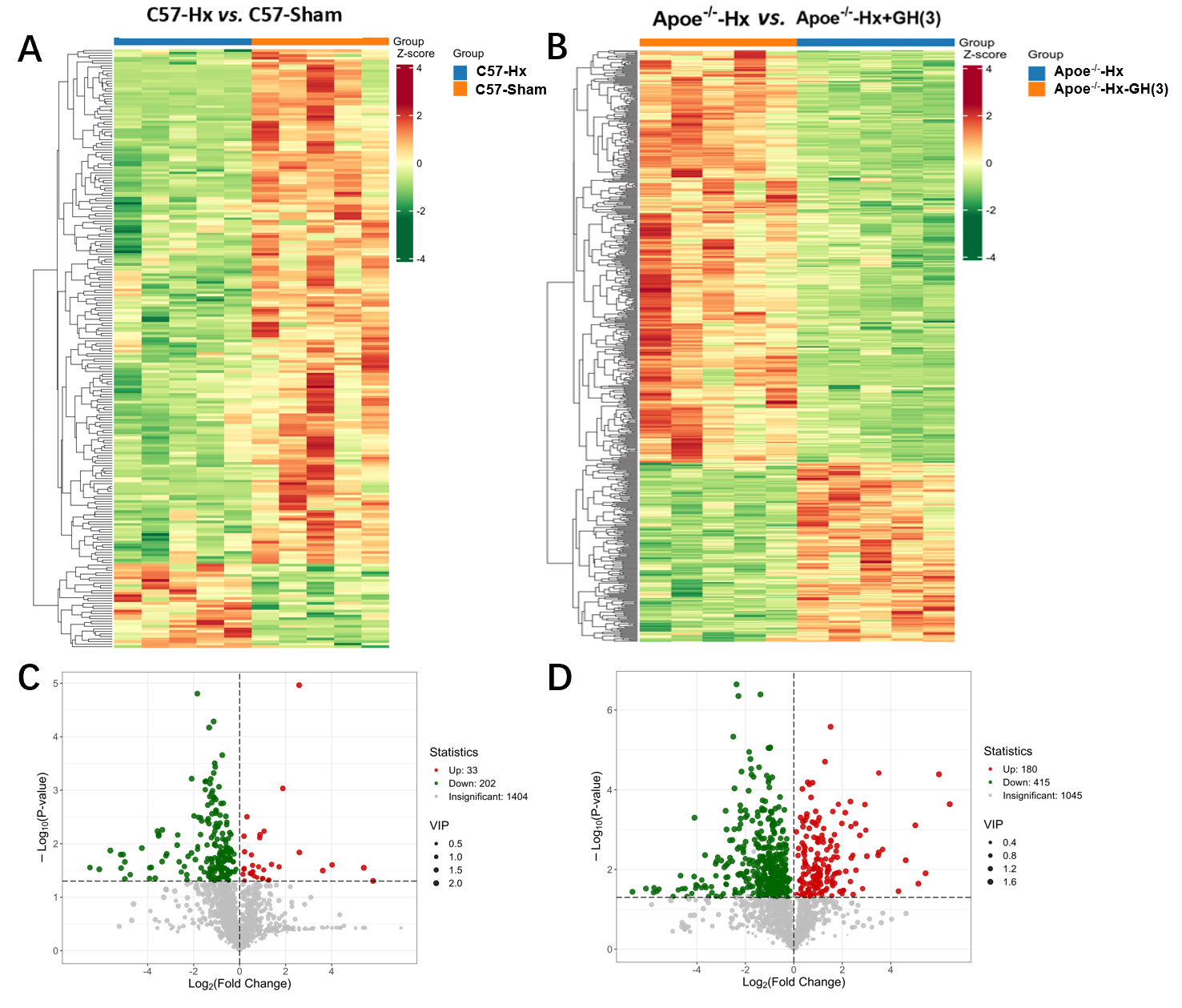


**Fig. S3 Pituitary removal caused differential expression of aortic vascular genes.**

(A) Gene expression Heatmap of abdominal aorta and control vessels in pituitary depleted mice detected by RNA sequencing (n=3). (B) Volcano plot of differentially expressed genes in the abdominal aorta and control vessels of pituitary depleted mice (n=3).


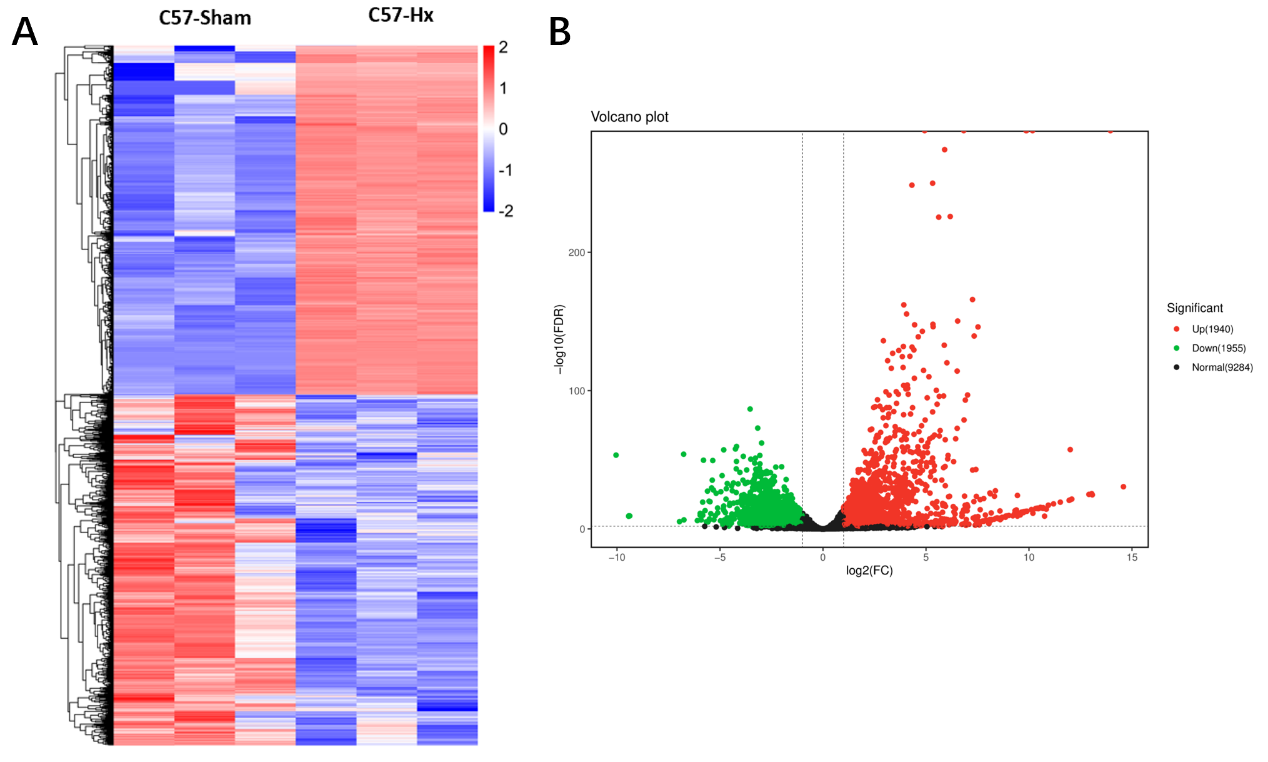

Supplement: Supplementary file 1 — Supporting Information Additional supporting information can be found online in the Supporting Information section. Figure S1: Comparative analysis of cell type composition in the aortas of atherosclerotic and control mice based on scRNA‐seq data. (a) Determination of marker genes for distinct cell populations in the mouse aorta for single‐cell analysis. (b, c) Comparative cell type profiling of aortas from atherosclerotic and control mice. Figure S2: GH induced differential lipid metabolism in mice. (a) Heatmap of serum metabolites in hypophysectomized mice and control mice detected by LC‐MS (n = 5). (b) Heatmap of serum metabolites in serum of GH‐treated ApoE−/−‐Hx mice and control ApoE−/−‐Hx mice detected by LC‐MS (n = 5). (c) Volcano plot of differential metabolites in hypophysectomized mice and control mice (n = 5). (d) Volcano plot of differential metabolites in serum of GH‐treated ApoE−/−‐Hx mice and control ApoE−/−‐Hx mice (n = 5). Figure S3: Pituitary removal caused differential expression of aortic vascular genes. (a) Gene expression heatmap of abdominal aorta and control vessels in pituitary depleted mice detected by RNA sequencing (n = 3). (b) Volcano plot of differentially expressed genes in the abdominal aorta and control vessels of pituitary depleted mice (n = 3). [file IJOG-2025-9710652-s001.docx]
